# Predicting Caller Type From a Mental Health and Well-Being Helpline: Analysis of Call Log Data

**DOI:** 10.2196/mental.9946

**Published:** 2018-06-11

**Authors:** Alexander Grigorash, Siobhan O'Neill, Raymond Bond, Colette Ramsey, Cherie Armour, Maurice D Mulvenna

**Affiliations:** ^1^ School of Computing Ulster University Newtownabbey United Kingdom; ^2^ School of Psychology Ulster University Coleraine United Kingdom

**Keywords:** data mining, machine learning, clustering, classification, mental health, suicide

## Abstract

**Background:**

This paper presents an analysis of call data records pertaining to a telephone helpline in Ireland among individuals seeking mental health and well-being support and among those who are in a suicidal crisis.

**Objective:**

The objective of our study was to examine whether rule sets generated from decision tree classification, trained using features derived from callers’ several initial calls, could be used to predict what caller type they would become.

**Methods:**

Machine learning techniques were applied to the call log data, and five distinct patterns of caller behaviors were revealed, each impacting the helpline capacity in different ways.

**Results:**

The primary findings of this study indicate that a significant model (*P*<.001) for predicting caller type from call log data obtained from the first 8 calls is possible. This indicates an association between callers’ behavior exhibited during initial calls and their behavior over the lifetime of using the service.

**Conclusions:**

These data-driven findings contribute to advanced workload forecasting for operational management of the telephone-based helpline and inform the literature on helpline caller behavior in general.

## Introduction

Telephone support services, crisis lines, and mental health helplines are provided in several countries as a way of addressing social and mental health problems, including loneliness. They are also provided as a means of supporting people who are suicidal and helping people access appropriate treatments. Additionally, they feature in suicide prevention antistigma campaigns, whereby people are encouraged to disclose mental health problems and seek help [1]. Although helplines are often the key elements of mental well-being and suicide prevention strategies, little is known about how these services are used. The analysis of call patterns and caller behavior data can help us understand how helplines are used and may also help understand more about the role of these services and the needs of the client groups who use them. Connectedness is an important feature of the models of suicidal behavior [2]. Seeking support can mitigate the effects of stress, and contacting a supportive listener may assist a person in overcoming suicidal thoughts and feelings [3,4].

This study involves the analysis of digital telephony data from Samaritans Ireland, a charity organization with a helpline to provide emotional support to anyone in distress or at risk of suicide. Although the organization offers support via short message service text messaging, email, and face-to-face, 95.0% of their contacts remain via telephone [5]. Data were provided for all calls made to the organization in the Republic of Ireland for almost a 4-year period (April 2013 to December 2016), comprising a total of 3.449 million calls. This amounts to 725 calls per 1000 population in the Republic of Ireland.

A review of the literature indicated that caller behavior has been the subject of several studies. Research has classified callers as “one-off” or “repeat callers,” and studies have found that 3.0% of callers take up 47.0%-60.0% of the service capacity [6-10]. Furthermore, research on call demand has shown that calls to helplines peak at weekends and in the evening during weekdays [11].

Suicide prevention and mental health antistigma campaigns frequently encourage the use of helplines for individuals in a mental health crisis, and Helplines Partnership has identified a trend of year-on-year increasing demand in UK helplines [12]. Reports on multiple helplines have also described an increase in the complexity of calls received [13-15].

However, none of these cited studies are specific to caller behavior derived from a large telephony dataset. We could not find any study that incorporated the analysis of a large dataset over a prolonged period to provide an understanding of call patterns and caller behavior. This study extends previous research by analyzing a much larger dataset and also using data analytics or data mining techniques [6-10]. We aimed to predict the caller type based on call log data derived from their initial calls. We used the calling history of a caller identity (ID) and attempted to predict whether that caller would become prolific.

In the operational research literature, call centers are studied as queuing systems, with a view to optimize the processing of the stream of call arrivals by adjusting the number and service properties of human phone operators or automated agents [16]. From the queuing theory standpoint, the organization operates a human-staffed, multiple-server system that processes inbound calls only, has a null-length queue (an arriving call is either immediately answered or dropped), retries (a caller redials after meeting an engaged tone), and reconnects (conversations spanning more than one call).

Due to commercial and data protection issues, call center datasets usually carry no information to differentiate one caller from another; hence, most operational research studies work with overall call volume and other aggregate characteristics of a call stream and a call center.

This paper reports on successful interdisciplinary research between computer science and psychology researchers and also technically concentrates on using machine learning to automatically classify callers into caller types. For example, to investigate whether we can use initial call data to predict if a caller is prolific, the caller could be served to specially trained staff, allowing for an enhanced service.

In summary, this paper attempts to answer the following research questions: (1) Can rudimentary data from call logs be used to determine caller types associated with specific calling patterns? (2) Can early use of the service or data from initial calls be used to predict this caller type?

The objective was to build a machine learning system that would be trained using attributes or features derived from callers’ several initial calls to predict what caller type they would become.

## Methods

### Overview

The dataset used in this study comprised several million records of calls made to the mental health helpline, with data fields including a unique caller ID, the date and time of a call, and the duration of the call. Several attributes of the callers were used as a basis for clustering, which is a form of unsupervised learning where data are explored without any a priori knowledge provided. The number of calls, mean duration of call, and SD of a caller’s calls were all used as a basis for clustering. The clusters that emerged from this process were then used as a starting point to generate a model that could be used to predict caller behavior, if successful. The number of calls, mean duration of call, and SD of a caller’s calls were again used as a basis for the predictive model building process, generating rule sets that were evaluated using commonly used measures, including accuracy and specificity. The methods are discussed in more detail in the following subsections.

### Dataset

The dataset comprises numerous fields; however, only the following fields were used in this study: (1) the date–time stamp of the call arrival precise to the last second, (2) the answered flag meaning that the call was passed to a Samaritans Ireland volunteer, (3) the duration of the call (seconds), and (4) the unique caller ID.

Each record in the organization’s dataset carries a caller ID that uniquely enumerates the caller, while revealing no personally identifying information. These IDs are associated with most, but not all, calls (around 20% of the calls have the caller ID missing).

### Caller Types or Classes of Interest

In this study, we restricted our view to the calls that were accompanied by non-null caller IDs, flagged as answered, and showed a positive duration. The dataset contained a total of 1.387 million of such calls in 2013-2016, coming from 53,629 unique caller IDs. This restriction excluded engaged or dropped calls; a helpline volunteer has no information on the number of redials a caller had to make to get through. This also excluded a small minority of imperfect records, such as answered calls with zero durations or dropped calls with positive durations.

The data showed that only 20,527 callers made two or more answered calls to the organization. A majority of these (12,258) got through for the second time within 7 days of the first call. The interarrival time between the arrivals of the first and the second answered calls ranged from 2 s to nearly 44 months, with the median time being 26.32 h and mean time being 2.67 months. This demonstrates a tendency to call again soon if a conversation has to be continued. This also indicates that more than half of the caller population talks to the organization once only.

### Discerning Caller Types: Clustering

Cluster analysis involves grouping a set of objects (ie, in this case, callers based on selected attributes) in such a way that objects in the same group (called a cluster) are more similar to each other compared with those in other groups (clusters).

Callers were clustered using the following three caller attributes: (1) number of calls, (2) mean call duration in seconds, and (3) SD of call duration, also in seconds. We selected these attributes due to their explanatory power: the number of calls a person makes indicates his or her frequency of help-seeking behavior; the mean call duration indicates call length; and SD of call durations indicates a person’s variability and consistency in conversation length.

We used K-means clustering algorithm because it is the most widely used and established clustering algorithm in the unsupervised machine learning literature. The number of cluster centroids is a user-defined parameter. Using the elbow method, we discerned that 5 is a reasonably small number of clusters that would provide a reasonable resolution in terms of explained variability.

We performed an experiment with a smaller and a larger number of clusters. A 3-cluster solution yielded a substantial reduction in the explained variability as follows: ~54% with 3 clusters versus ~74% with 5 clusters. Attempts to build a solution with 6-11 clusters exacerbated the issues in explaining the ever-finer distinctions between the clusters, whereas the explained variability of the data increased only moderately, never exceeding 86%. The Elite Prolific cluster (callers who call several times) remained very stable throughout, and the largest Typical cluster (callers who call 5 or 6 times) remained more than 3.5 times as large as the second largest cluster. Overall, 5 clusters provided intuitively the best picture that is rich enough to be of interest to psychologists and simple enough to interpret.

### Predictive Classifier Algorithm

The predictive models used in this experiment were based on the C5.0 algorithm that works using decision trees or rule sets. This algorithm was used due to its reliability and success in classification problems [17]. Decision trees and rule sets form one of the pellucid techniques used in data mining as opposed to black-box techniques, such as artificial neural networks. An example of a model generated using C5.0 from our dataset is shown in the Discussion section.

### Models Cascade

Once we discerned the caller type of each caller by clustering the 4-year worth of calls to the organization, we approached the question of predicting the eventual caller cluster or type from a few initial observations of the caller. We were not interested in the callers who call only once. Their behavior is defined by their single answered call. Therefore, we concentrated on those who had at least two answered calls. There were 20,527 such callers. This spawned the following questions: how many calls from each caller would be required in order to be able to provide a prediction on caller type, and what would be a reasonable observation timespan within which to collect those initial calls?

To address these subquestions, we built a cascade of predictive classification models, for which the data were collected subject to a system of the following three conditions:

1. Condition 1: at most N calls. A model was to be built using the initial N calls by each caller. If a caller made less than the answered N calls, we collected that lower number of calls. The values of N cascaded through the following sequence: 2, 4, 8, 16, 24, 32, 48, 64, 256, 1024, 2048, 4096, and 8192. We hoped that low values of N would reasonably suffice overall. However, the decisive property of the Elite Prolific cluster was a high volume of calls made in any given time period (see the Results section). Therefore, we needed high values of N to view how our models fared at detecting the callers from the Elite Prolific cluster. The callers from other clusters made no more than 5,384 calls each; thus, any number of calls higher than that would unambiguously point to Elite Prolific cluster. This decided for us the sufficient ceiling of N to be at 8,192 calls.
2. Condition 2: observation timespan. The initial 2 to N calls per caller had to be collected within a restricted timespan starting from the arrival of the first call by that caller. We chose the following cascade of observation timespans: up to 7 days (1 week), up to 30 days, up to 120 days (4 nominal months), up to 52 weeks (1 business year), and (for completeness) an unrestricted timespan. It has to be emphasized that the observation timespan was calculated individually for each caller, starting at the arrival timestamp of the first call by that caller. This created a relative timescale that enabled capturing similar behavior of different callers, over, say, 30 days of observation, regardless of the individual starting points.
3. Condition 3: number of classes. We examined three different ways to predict the caller type. First, a 5-class model classified the callers according to the 5 caller types discerned from clustering. Second, a 3-class model classified Elite Prolific callers distinctly from Standard Prolific callers, while a class called Other labeled the remaining caller types. Finally, a 2-class model labeled the callers as either Prolific or Other, where the Prolific class comprised the callers from Elite Prolific and Standard Prolific clusters and the Other class comprised callers from Typical, Unpredictable, and One-Off clusters.

### Classification Features

Subject to the conditions listed in the previous section, for each model, we computed the following features associated with each individual caller ID: (1) Count (number of the calls actually collected out of the N calls allowed), (2) MeanDur (mean duration of these initial calls, in seconds), and (3) SDDur (SD of this duration, in seconds). This ensured that the per caller metrics computed for classification matched the metrics computed in clustering (see the section “Discerning call types: clustering” above).

Because we limited the number of initial calls fed to the classifier at N, it became important to enable the classifier algorithm to see the information related to the individual length of time actually taken by each caller to generate up to the requisite number of answered call “arrivals.” For example, 20 of the 40 Elite Prolific callers made their initial 4 calls within 2 h from the start of the first call. In contrast, for 3,192 Typical callers who actually made 4 or more calls, the median time to generate these 4 call arrivals was more than 164 h, or almost 7 days. The slowest 25% of callers of all types had generated their first 4 call arrivals in more than 19 days.

For our predictive models to account for this variation in the individual call arrival dynamic, we introduced a feature in addition to the above-listed three features. The new feature was named IATLife, Inter-Arrival-Time “Life,” and computed as the number of seconds between the first and the last call arrival of those initial count calls.

Altogether, every one of the 195 classification variants was performed against the following features: Count, MeanDur, SDDur, and IATLife.

### Evaluation Metrics and Cross-Validation

A suite of conventional metrics was used to evaluate the decision tree, including accuracy, kappa, sensitivity, and specificity. To avoid accuracy paradoxes, significance tests of accuracy rates against the no-information rate (NIR) were conducted (where *P*<.05 equates a significant model that has some value). NIR is simply the proportion of the most popular caller type or class in the dataset.

We specified a 4-fold cross-validation to produce reliable evaluation metrics, instead of the traditional 10-fold cross-validation. We chose 4-folds because we had only 40 Elite Prolific callers (see Table 1). A 75:25 hold-out split reserves 10 of them for testing and leaves 30 of them for training. When training with 4-fold cross-validation, each fold receives between 7 and 8 Elite Prolific callers. If the training is done with the default 10-fold cross-validation, each fold would receive fewer than 5 Elite Prolific callers, making them look like outliers among the mass of callers.

Our decision to simplify cross-validation left confusion matrices practically unchanged; data for 16 initial calls are presented in Table 2.

Performance metrics did not show any noticeable change either; see Table 3.

In all, using a 4-fold cross-validation detracted nothing from the models’ reliability, while improving the speed of training by a factor of more than 2.5.

### Software

R programming language and R Studio were used for data wrangling and to implement the analysis. R libraries were used namely dplyr, readr, tibble, tidyr, scales, and DescTools for wrangling; ggplot2 and DescTools for generating the visuals; fpc and cluster for clustering diagnostics; and caret and C50 for classification, while the base package stats provided routines for K-means clustering.

**Table 1:** Clustering results showing cluster averages for each of the three features as well as cluster sizes and values of the within-cluster sum of squares, using 5-cluster data for years 2013-2016 and explained variability of 73.77%.

Name	Volume	Mean	SD	Size	Within_SS^a^
ElitePro^b^	11042.03	194.60	380.60	40	7481.88
Typical	4.80	314.61	33.71	35218	7395.90
StandPro^c^	88.71	944.70	737.24	7895	12656.26
Unpredic^d^	24.66	1886.14	1605.73	2727	5890.24
OneOff^e^	1.16	2162.42	32.15	7749	8780.66

^a^Within_SS: within-cluster sum of squares.

^b^ElitePro: Elite Prolific callers.

^c^StandPro: Standard Prolific callers.

^d^Unpredic: Unpredictable erratic callers.

^e^OneOff: One-off chatty callers.

**Table 2:** Confusion matrices.

Prediction		Reference		
			Prolific	Other
**4-fold cross-validation**				
	Prolific		1869	70
	Other		114	3078
**10-fold cross-validation**				
	Prolific		1886	75
	Other		97	3073

**Table 3:** Performance metrics for 16 initial calls.

Cross-validation	Acc^a^	95% CI	NIR^b^	*P* value (Acc > NIR)	Kappa	Sens^c^	Spec^d^	PPV^e^	NPV^f^	F1^g^	Prevalence	Detection rate	Detection prevalence	Balanced accuracy
4-fold cross- validation	0.96	(0.96- 0.97)	0.61	<.001	0.92	0.94	0.98	0.96	0.96	0.95	0.39	0.36	0.38	0.96
10-fold cross- validation	0.97	(0.96- 0.97)	0.61	<.001	0.93	0.95	0.98	0.96	0.97	0.96	0.39	0.37	0.38	0.97

^a^Acc: accuracy.

^b^NIR: no-information rate.

^c^Sens: sensitivity.

^d^Spec: specificity.

^e^PPV: positive predictive value.

^f^NPV: negative predictive value.

^g^F1: F1 score.

## Results

The existence of different caller types was initially suggested by observed distribution of call durations. For example, fitting simple distribution curves (e.g., negative exponential, gamma, etc) to this distribution revealed the presence of a systematic variation in the residuals, which was suggestive of a stratified nature of the underlying population of callers.

### Caller Types Emerged From Clustering

**Figure 1:**
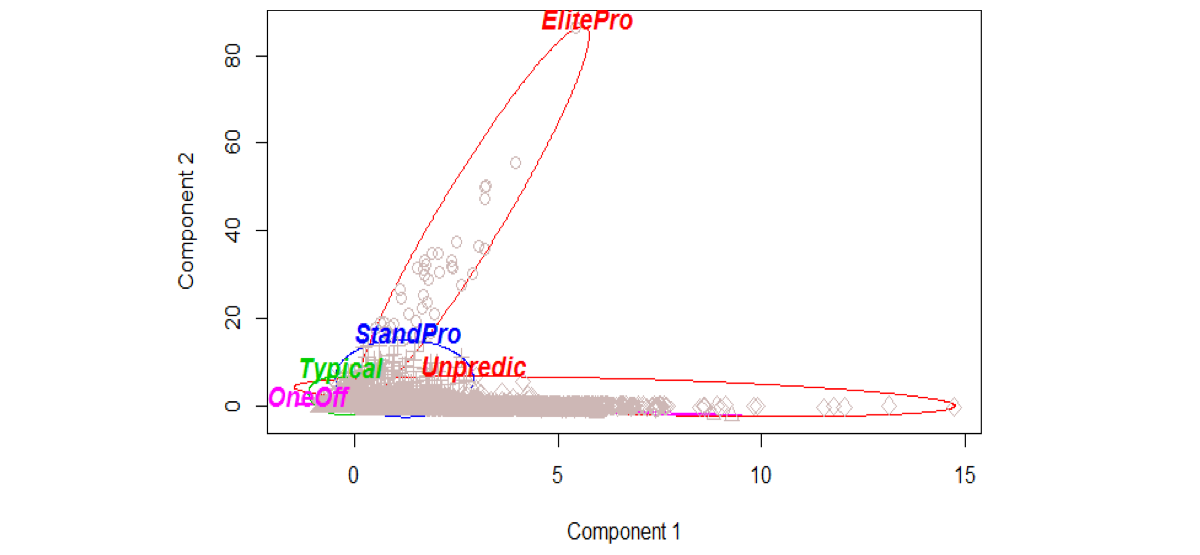
Clustering results, showing a projection from the feature space onto the plane of two principal components. These two components explain 77.54% of the point variability.

Table 1 and Figure 1 show the clusters (caller types) and their properties for a 5-cluster split using the entire 2013-2016 data timespan. The properties are as follows:

1. Name: see the interpretation below.
2. Volume: in-cluster mean of the number of calls made from each caller within that cluster.
3. Mean: in-cluster mean of the personal mean duration (seconds) of calls within that cluster.
4. SD: in-cluster mean of SD of call durations (seconds) from callers in that cluster.
5. Size: cluster size; number of callers captured in the cluster.
6. Within_SS: sum of squares characterizing the dissimilarity of callers captured within this cluster. The smaller this number, the more the homogeneity exhibited in the cluster.

We interpreted the 5 clusters shown in Table 1 as follows:

1. Cluster “ElitePro” (Elite Prolific callers): the largest average number of calls per caller in the cluster and the smallest cluster size. These were a handful (fewer than 50 callers over the 4-year timespan) of extremely prolific callers responsible for 20.0% of the total call volume. They called thousands of times, with half the call durations not exceeding 2.5 min and a small minority of calls lasting about 10 min.
2. Cluster “Typical” (Typical callers): the largest cluster size. These were the majority of callers who called 1-5 times and, in most cases, had a short 2.5- to 9-minute conversation. This cluster comprised about 66.0% of all the callers.
3. Cluster “StandPro” (Standard Prolific callers): second largest average number of calls per caller, middling average call duration and the largest unexplained variability encompassed by the cluster. About 15.0% of the callers were prolific, each making half a dozen to several dozens of calls and generating call durations that were moderate in length (10 min to half an hour long).
4. Cluster “Unpredic” (Unpredictable erratic callers): the largest average SD of the call duration. These were about 5.0% of the callers whose call duration varied considerably, with some calls lasting 9 min and some over 1.5 h.
5. Cluster “OneOff” (One-off chatty callers): the smallest average number of calls per caller accompanied by the largest average call duration. These were about 14.0% of the callers who called only 1-2 times; most had a lengthy 30-min- to 1-h-long conversation and did not return for any sustained support. These were the operational opposite to prolific caller clusters. One exceptional conversation in this cluster lasted for 3.44 h, the absolute record of duration in the dataset.

Principal component axes form a plane in the feature space oriented such that the projection of the scaled dataset onto this plane shows the widest possible 2-dimensional footprint of the dataset. The feature space in this case is 3-dimensional because we cluster with the values of the following 3 numeric features: volume, mean, and SD.

We experimented with the robustness of the emerged clusters against time slicing. The 5-cluster split was first done for the 2013-2015 timespan, then for the 2013-2016 timespan, and then rerun for each of the years 2013, 2014, 2015, and 2016, separately. In all these time slices, the prominent features of the identified clusters remained invariant. The graphical images of the clusters in terms of the principal components retained their visual features, such as the shape and the relative position in the feature space of each cluster. For example, the Elite Prolific cluster in each case produced a shape that dictated the direction of the second principal component axis; refer to the image of cluster ElitePro in Figure 1.

The “explained variability” in Table 1 and Figure 1 is the ratio of the amount of variability explained by clustering to the total amount of variability in the dataset. The statistical variability is measured in terms of the sums-of-squares of deviation of individual observations from the cluster center point. In all of our reruns of the 5-cluster split, the variability explained by clusters fluctuated at slightly under 74%, which was marginally lower than the variability explained by the principal components.

Notably, the caller distribution to classes was quite unbalanced; see the column “size” in Table I. A no-information model that always classifies new cases into the dominant class (“Typical” cluster in our case) would be correct 66% of the time ([35,218/53,629] × 100). Therefore, the predictive ability of the developed models needs to be, at minimum, better than 66% and, ideally, much higher.

### Classification Results

Altogether, we had 13 ways to specify the number of calls, 5 ways to specify the observation timespan, and 3 types of class specification, as described above in Models Cascade under Methods section. In total, the cascade contained 195 variants of our predictive model.

For brevity of description, in what follows, we will be using a Whyte-like notation to refer to a specific model: L-N-T, where L shows the number of target classes, N shows the maximum number of calls collected per caller, and T specifies the observation timespan. For example, the expression “2-16-30D model” means “2-class model built with up to 16 calls collected within the first 30 days of observation of each caller.” The expression “5-1024-U model” means “5-class model built with up to 1024 initial calls and unrestricted timespan.” The expression “2-*-52W models” denotes a collection of all 2-class models with a 52-week (1 nominal year) observation timespan for every number N of initial calls examined; see Condition 1 above.

#### 2-*-U Models

**Figure 2:**
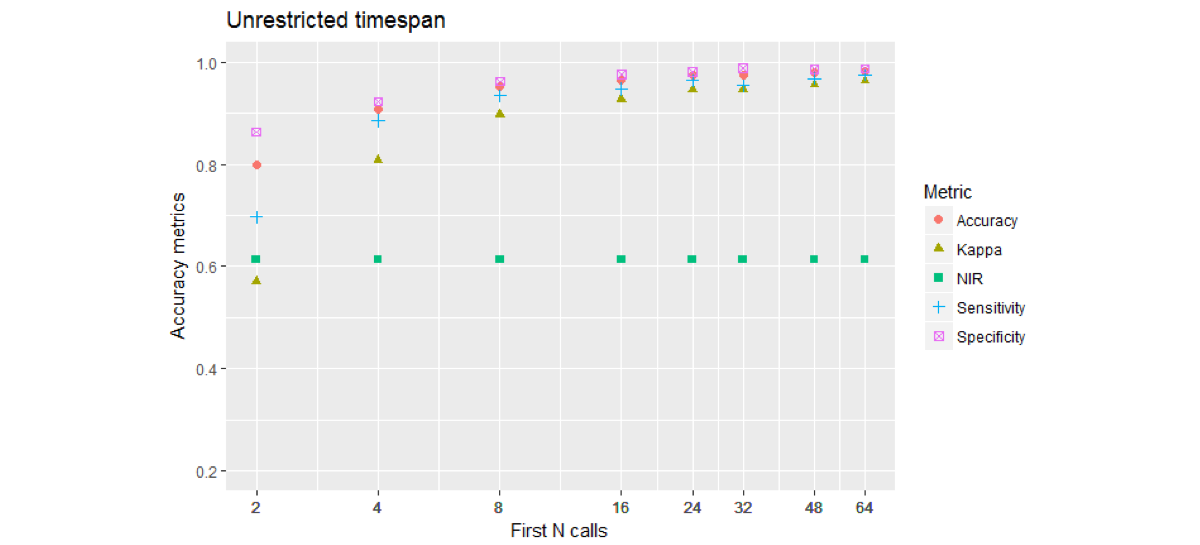
Performance of the 2-class models. The 4 performance metrics reach or exceed 0.9 (90%) for 8 calls and above, exceed 0.95 (95%) for 64 calls.

Figure 2 shows the results based on a model to predict Prolific callers distinctly from all the Others.

#### 3-*-U Models

**Figure 3:**
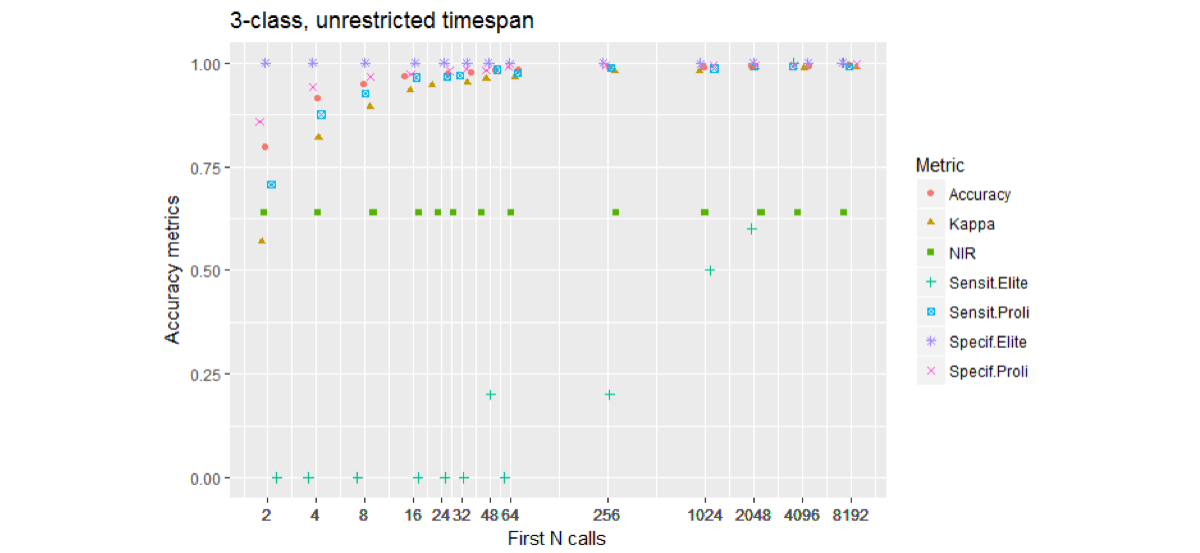
Performance of the 3-class models. Accuracy and Kappa are performance metrics for the model overall. Sensit.Elite and Specif.Elite are the sensitivity and specificity for the Elite Prolific class. Sensit.Proli and Specif.Proli are the sensitivity and specificity for the Standard Prolific class. Notice that the model begins to sense Elite Prolific callers as a separate class when fed over 200 initial calls, and about 4000 initial calls are needed to detect Elite callers reliably (both sensitivity and specificity are close to 100%).

Figure 3 shows the results of a model to predict one of the three classes or caller types.

The key observation is that the 3-class models are mildly sensitive to Elite Prolific callers—picking up some from 32 calls on, notably so from more than 200 calls on, and picking all of them from 4096 calls on. NIR, that is, the prevalence of the dominant Other class, was 0.61 for this group of models. An example confusion matrix, for the 3-1024-U model, is shown in Table 4.

The sensitivity for Elite Prolific callers reached 0.5; furthermore, 5 of the 10 such callers were predicted correctly.

#### 5-*-U Models

**Figure 4:**
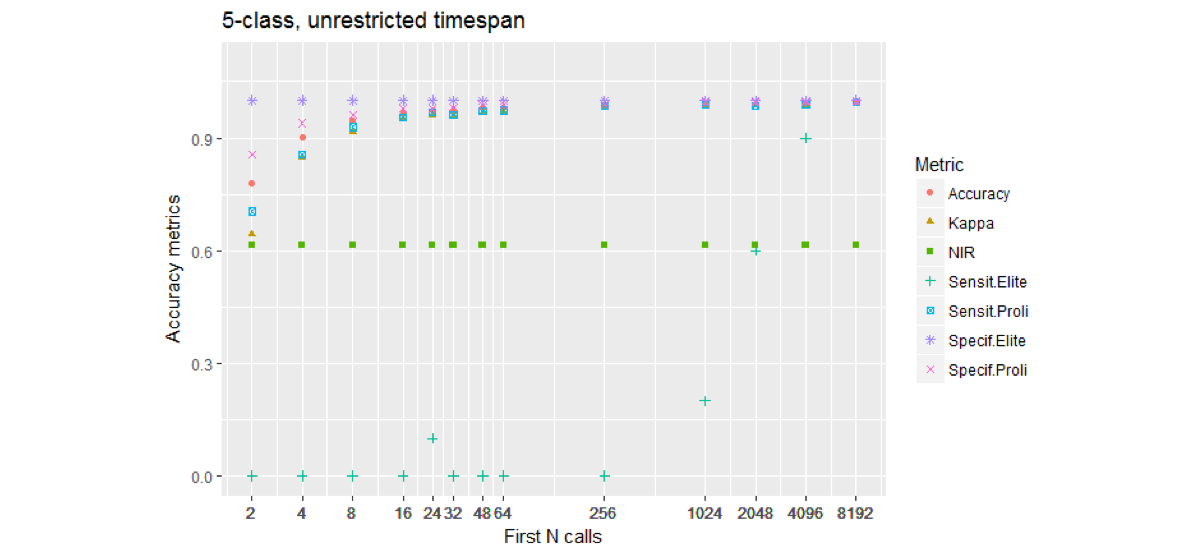
Performance of the 5-class models. Accuracy and Kappa are performance metrics for the model overall. Sensit.Elite and Specif.Elite are the sensitivity and specificity for the Elite Prolific class. Sensit.Proli and Specif.Proli are the sensitivity and specificity for the Standard Prolific class. The model needs over 1000 initial calls to be able to begin distinguishing Elite Prolific callers from others.

Figure 4 shows the results of a model to predict one of the 5 caller types.

The sensitivity and specificity values are plotted for the Elite Prolific class and Standard Prolific class, but not for the remaining 3 classes. The NIR, that is, the prevalence of the dominant Typical class, was 0.44 for this group of models. An example confusion matrix, for the 5-1024-U model, is shown in Table 5.

**Table 4:** Confusion matrix for the 3-1024-U model.

Prediction	Reference		
	ElitePro	StandPro	Other
ElitePro^a^	5	7	00
StandPro^b^	4	1947	16
Other	1	19	3132

^a^ElitePro: Elite Prolific callers.

^b^StandPro: Standard Prolific callers.

**Table 5:** Confusion matrix for the 5-1024-U model.

Prediction	Reference				
	ElitePro	StandPro	Typical	Unpredic	OneOff
ElitePro^a^	2	5	1	0	0
StandPro^b^	8	1949	9	3	0
Typical	0	5	2268	0	0
Unpredic^c^	0	9	0	678	0
OneOff^d^	0	5	1	0	187

^a^ElitePro: Elite Prolific callers

^b^StandPro: Standard Prolific callers

^c^Unpredic: Unpredictable erratic callers.

^d^OneOff: One-off chatty callers

The sensitivity for Elite Prolific callers remained at 0.2; only 2 of the 10 such callers were predicted correctly.

Notably, despite being more balanced in terms of class proportions than the abovementioned 3-class models, the 5-class models tended to perform less well at correctly identifying Elite Prolific callers; the Sensit. Elite values plotted in Figure 4 increase slower than the respective values shown in Figure 3.

It has to be emphasized that the 5- and 3-class models discussed in this and previous subsections were built without any limit to the call collection timespan. That is, to pick out Elite Prolific callers, one must be willing to collect arrival timestamps and durations of thousands of calls per caller, as well as be prepared to wait for quite a long time, the total timespan for this dataset being 45 months.

As soon as we began training models with restricted timespans, we found that the ability of both the 3- and 5-class models to predict Elite Prolific callers diminished quickly. Whether such a time-constrained observation of a caller provides enough statistical evidence to tell the difference between the Elite Prolific type and other types remains to be investigated.

We then moved on to investigate how well can our approach tell apart Prolific callers in general from Other callers in general under the condition of a restricted timespan. The results are shown in the next subsection.

#### 2-Class Models With Restricted Timespan

**Figure 5:**
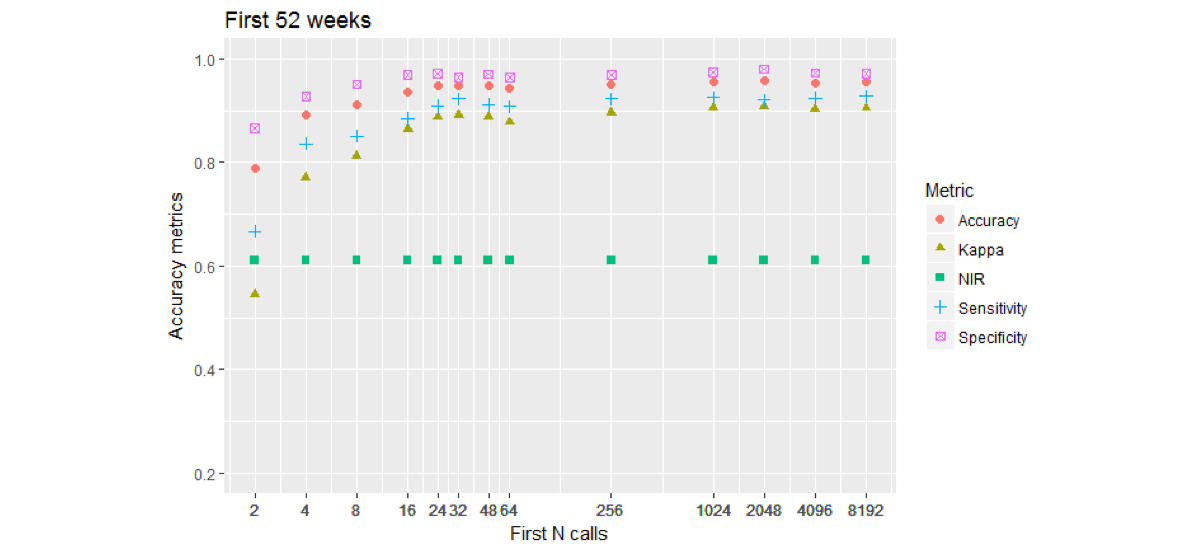
Performance of the 2-class model. Call collection timespan is restricted to 52 weeks (364 days) from the first call. Accuracy metrics reach or exceed 0.8 (80%) for 8 calls and above, it takes over 200 calls to lift the Kappa metric to 0.9 (90%).

Figure 5 depicts the results shown by the 2-*-52W models.

Compared with the unrestricted 2-*-U models, shown in Figure 2, about 10% of accuracy was lost by all metrics for 8 initial calls.

**Figure 6:**
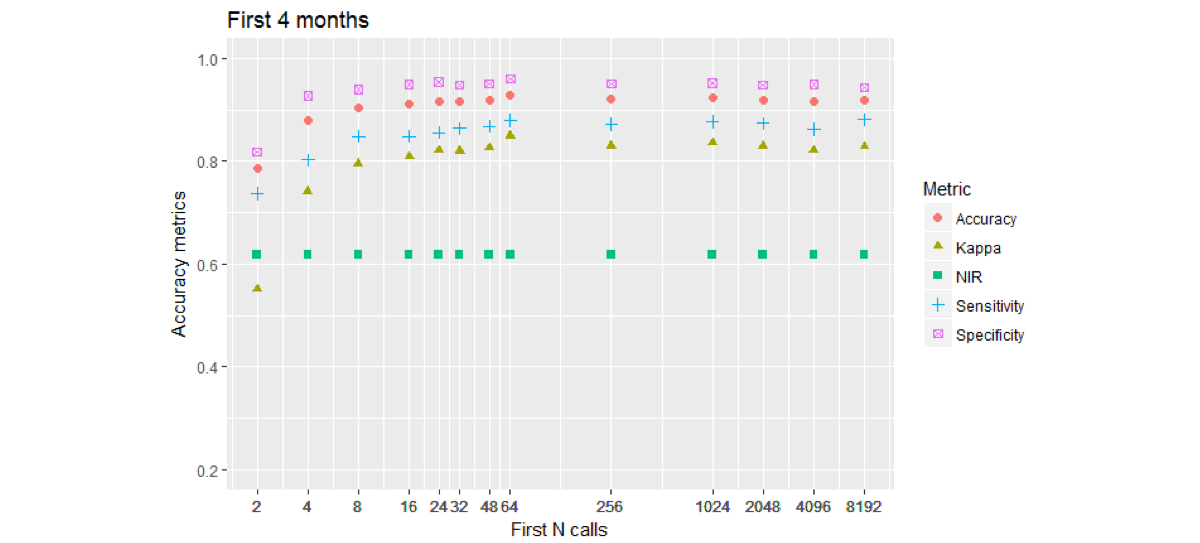
Performance of the 2-class model. Call collection timespan is restricted to 4 months (120 days) from the first call. Accuracy metrics reach or exceed 0.8 (80%) for 8 calls and above. Neither Sensitivity nor Kappa metrics reach values over 0.9 (90%).

Figure 6 shows the 2-*-120D model results (timespan of 4 months).

Accuracy limitations became apparent: Kappa and sensitivity reached a ceiling below 0.9. These metrics reached their ceiling between 80%, for kappa, and 95%, for specificity, when the model was built using between 8 and 16 initial calls. Sampling more calls showed virtually no effect on performance metrics.

**Figure 7:**
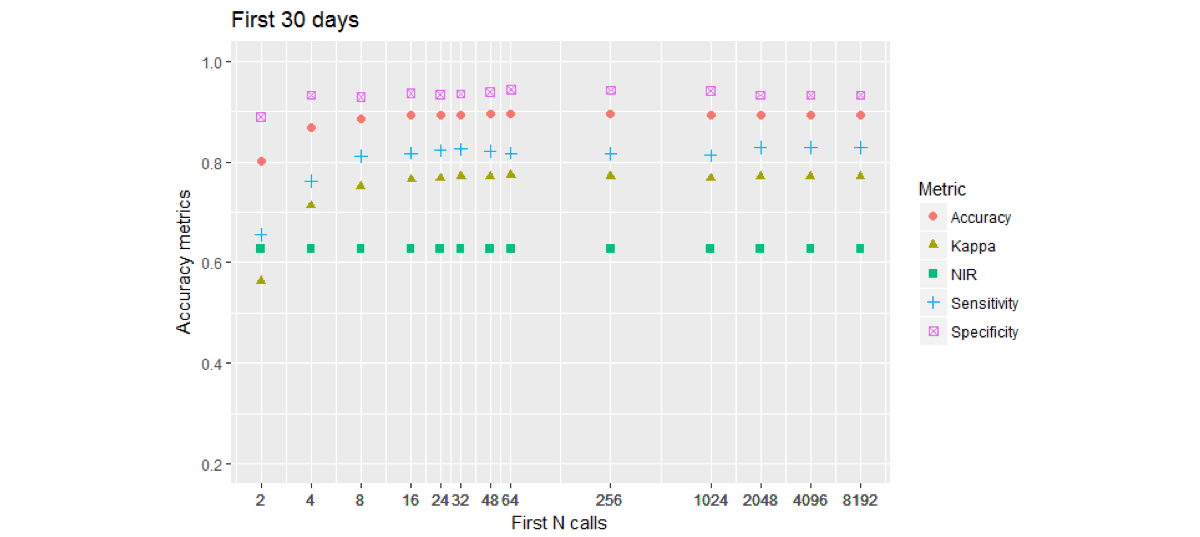
Performance of the 2-class model. Call collection timespan is restricted to 30 days from the first call. All accuracy stabilize at the levels they have achieved with initial 8 to 16 calls. Kappa no longer reaches 0.8 (80%) level.

Figure 7 shows the 2-*-30D model results (timespan of 1 month).

Metrics reached their ceilings and stayed at the levels they achieved when the model was built using 8-16 initial calls. Kappa reached a ceiling at 75%, etc. It was remarkable that reducing the watching time 4-fold—from 120 down to 30 days—resulted in a rather small reduction of performance. This means that in most cases, caller behavior becomes apparent within the first month of their contact with the organization and the caller type can be inferred from the initial 8 calls or so.

**Figure 8:**
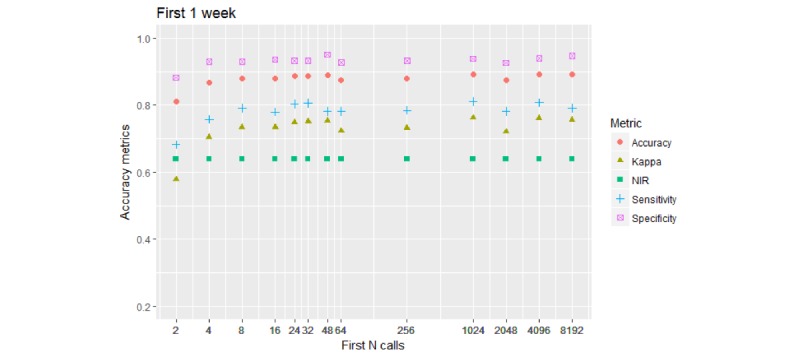
Performance of the 2-class model. Call collection timespan is restricted to 1 week (7 days) from the first call. All accuracy stabilizes at the levels they have achieved with initial 8 to 16 calls. Kappa fluctuates at mid-70% level. Sensitivity fluctuates around 80% mark.

Figure 8 shows the 2-*-7D model results (timespan of 1 week).

Kappa was firmly below 80%; its value stabilized when callers were classified using between 8 and 16 initial calls.

The results showed that we can obtain a decent accuracy of predicting the binary Prolific/Other caller type when using attributes from the first 8 calls and the first 30 days—whichever comes first, that is, the 2-8-30D model. Decision rules for this scenario are shown in Table 6, where attribute usage was 100.00% for standard deviation of initial calls (SDDur), 71.38% for mean duration of initial calls (MeanDur), 17.27% for Count; and 14.25% for Inter-Arrival-Time Life (IATLife).

This decision rule set consists of 16 rules. Each rule comprises one or more antecedents that must be fulfilled for the rule to trigger, along with the class predicted by this rule, and the confidence value of the class prediction shown in square brackets and ranging from 0 to 1. Each rule is accompanied by an (n/m) expression, where n shows the number of training cases that trigger this rule and m shows how many of those n cases are misclassified by this rule. Several rules, with possibly conflicting predictions, may be applicable to classify a new case. To decide the final predicted class value, the applicable rules vote for their predictions with the voting weight equal to the confidence value reported. The votes are accumulated, and the predicted class with the highest total vote is chosen as the result. If none of the rules apply, the Default class is chosen.

For example, a case of a caller comprising 6 calls with MeanDur equal to 600 s and SDDur equal to 330 s triggers Rule 2, Rule 8, and Rule 14, with accumulated votes resulting in the predicted class Prolific.

The attribute usage statistics reproduced at the bottom of Table 6 for each of our features show values in excess of 10%, confirming that every feature should be kept, and the C5.0 algorithm has not done feature reduction (despite attempting to do so by default).

The confusion matrices obtained upon testing this 2-8-30D model on the caller data it has not seen during training are shown in Table 7.

**Table 6:** C5.0 decision rules for the 2-8-30D model.

Rule number (training cases applicable/misclassified)	Class predicted (confidence)	Antecedents
Rule 1 (516/26)	Prolific (0.948)	Count ≤ 7
		MeanDur^a^ > 646.875
		MeanDur ≤ 837
		SDDur^b^ > 391.805
		SDDur ≤ 1290.503
Rule 2 (367/23)	Prolific (0.935)	MeanDur > 549
		MeanDur ≤ 646.875
		SDDur > 386.0335
Rule 3 (169/11)	Prolific (0.930)	MeanDur > 837
		MeanDur ≤ 1632.125
		SDDur > 320.4684
		SDDur ≤ 433.749
Rule 4 (120/8)	Prolific (0.926)	Count > 7
		MeanDur > 646.875
		MeanDur ≤ 837
		SDDur > 320.4684
		SDDur ≤ 1290.503
Rule 5 (269/39)	Prolific (0.852)	Count > 7
		MeanDur > 930.5
		MeanDur ≤ 1678.5
		SDDur > 320.4684
		SDDur ≤ 1810.193
		IATLife^c^ ≤ 2420253
Rule 6 (332/51)	Prolific (0.844)	MeanDur > 776.125
		MeanDur ≤ 1575
		IATLife > 1886978
Rule 7 (96/15)	Prolific (0.837)	MeanDur > 930.5
		MeanDur ≤ 2126.5
		SDDur > 320.4684
		SDDur ≤ 1810.193
		IATLife > 2420253
Rule 8 (5432/1917)	Prolific (0.647)	SDDur > 320.4684
Rule 9 (4703/310)	Other (0.934)	MeanDur ≤ 353
		SDDur ≤ 464.1454
Rule 10 (806/97)	Other (0.879)	Count ≤ 7
		SDDur > 1376.03
Rule 11 (664/80)	Other (0.878)	Count ≤ 3
		MeanDur > 1124
		SDDur > 1197.839
Rule 12 (63/7)	Other (0.877)	Count > 7
		MeanDur > 930.5
		SDDur > 1290.503
		IATLife > 456108
		IATLife ≤ 2420253
Rule 13 (564/69)	Other (0.876)	MeanDur > 1716
		SDDur > 1124.133
Rule 14 (6178/855)	Other (0.861)	SDDur ≤ 433.749
Rule 15 (990/139)	Other (0.859)	MeanDur > 930.5
		SDDur > 1290.503
		IATLife ≤ 2420253
Rule 16 (590/102)	Other (0.826)	MeanDur > 2062.667
		SDDur > 320.4684
Default	Other	

^a^MeanDur: mean duration of initial calls

^b^SDDur: standard deviation of initial calls.

^c^IATLife: Inter-Arrival-Time Life

**Table 7:** Confusion matrices for 2-8-30D rule set model.

Prediction	Reference	
	Prolific	Other
Prolific	1057	113
Other	287	2147

## Discussion

### Overview

The use of machine learning can provide crisis support and suicide prevention helplines with the knowledge of a caller’s behavior patterns, generally, and the knowledge of what caller type any individual is likely to become, specifically, based on his or her initial behavior. This may be useful for providing basic business intelligence for operational management or for simply internally signposting callers to specialized teams who may be better placed to offer them the support they require. However, as with most machine learning tasks, there is a question of ethics. For example, one must consider the impact of falsely allocating an individual to an inappropriate service or failing to recognize patterns associated with an increased risk of suicide. The greater concern is predicting a caller to become a Prolific caller and signposting him or her to a specially trained team when in fact the caller is actually a Typical caller.

### Future Work

Our future work will involve using other machine learning techniques such as artificial neural networks and support vector machines to determine the best performing model. Decision rules were used here due to their transparency and to obtain a benchmark for future studies.

Figuring out whether a restricted observation timespan, of say 30 days, is sufficient to detect statistical differences between Elite Prolific callers and the callers of other types is an important future issue that would determine the possibility of success at early detection of the rare, but influential, Elite Prolific caller type.

Early identification of Elite Prolific caller type and routing the calls of these callers to specialized advisers informs the modeling of health care service usage, offering insights for evidence-based practice and operational decision making.

### Limitations of This Study

The dataset is anonymous and uses a unique identifier to determine repeat callers. The limitation is that this identifier is based on a phone number, and although there is no corroborating evidence of multiple users of a single phone number from the helpline provider, the possibility exists that there could be more than one service user using the same phone line. The analysis is partly limited by not filtering the data based on caller tenure and recency. For example, callers may be ending their tenure at the start of our observable dataset time window and other callers may be starting their tenure at the end of our window. This results in some misclassifications; for example, Prolific callers ending their tenure at the start of our window could be misclassified as Typical callers. However, this limitation is mitigated given the length of the observable time window in our dataset. This is evidenced by the fact that we achieve a high accuracy rate for classification caller types. We would, however, recognize that higher accuracy rates may be achieved by filtering the caller dataset based on the each caller’s exposure of their tenure.

### Conclusions

This work shows that a significant model (*P*<.001) for predicting caller type from the data obtained from the first ~8 or ~16 calls can be achieved. For example, Table 3 indicates that the accuracy of the model was 96.41%, which is +35.06% greater than NIR (61.35%). This indicates an association between caller behavior exhibited in the initial calls and their behavior over the lifetime of using the service.

Additionally, this work shows that one can model the different types of service users (callers) and the complex nature of caller behavior and patterns to optimize resource management, volunteer productivity, and forecast demand. The groups of callers may have different levels of risk in relation to mental health and suicide, which may then direct the care required. For example, the callers who use the service for a short period of time during a crisis or a recurrence of mental illness might be managed differently from those prolific callers who may be at risk of overdependence on the service. Further analysis to understand the needs of these groups, their demographic profile, and the topics discussed would, therefore, be of value. This analysis offers an opportunity to review the skillset and the training needed by volunteers to best support service users. Matching skillsets and training to caller needs serves to improve job satisfaction and productivity.

Data mining of the association of caller IDs with the volume and duration of calls revealed several caller clusters, each describing a distinctive behavior type. The most striking of these clusters, termed Elite Prolific callers by us, encompassed a small number of caller IDs responsible for a substantial share of the total call volume that Samaritans Ireland received. Further research is needed to identify whether the identified caller groups have specific types of mental health and support needs to inform staff training and caller management guidance.

## Abbreviations

IATLife: Inter-Arrival-Time Life
ID: identity
MeanDur: mean duration of calls
NIR: no-information rate
SDDur: SD of duration
